# Caseous Mitral Annular Calcification Causing Left Ventricular Outflow Tract Obstruction and Embolic Stroke in Hypertrophic Cardiomyopathy

**DOI:** 10.1016/j.jaccas.2026.108917

**Published:** 2026-06-18

**Authors:** Valentina Bogini, Federica Carusone, Andreina D'Agostino, Giovanni Benedetti, Fausto Pizzino, Giancarlo Trimarchi, Michele Murzi, Maria Laura Canale, Sergio Berti, Massimiliano Mariani

**Affiliations:** a Interdisciplinary Research Center for Health Science, Sant’Anna School of Advanced Studies, Pisa, Italy; b Department of Advanced Biomedical Sciences, Federico II University of Naples, Naples, Italy; c Cardiology and Cardiovascular Medicine Department, Fondazione Monasterio, Massa, Italy; d Cardiology and Cardiovascular Medicine Department, Ospedale Versilia, Camaiore, Italy

**Keywords:** cardiomyopathy, echocardiography, imaging, mitral valve, rheumatic heart disease, secondary prevention, stroke, three-dimensional imaging, valve replacement

## Abstract

**Background:**

Caseous mitral annular calcification is a variant of annular calcification that may alter mitral valve geometry and represent a potential embolic source.

**Case Summary:**

A 50-year-old woman with exertional dyspnea was diagnosed with hypertrophic cardiomyopathy with left ventricular outflow tract obstruction. Transesophageal echocardiography revealed caseous calcifications of the posterior mitral annulus, causing leaflet displacement, promoting systolic anterior motion. Despite optimized medical therapy, she developed ischemic stroke, with imaging suggesting liquefactive degeneration of the masses as a potential embolic source.

**Discussion:**

This case highlights a mechanistic link between mitral annular calcification and left ventricular outflow tract obstruction. Caseous mitral annular calcification may act as an embolic source following liquefactive degeneration.

**Take-Home Messages:**

Caseous mitral annular calcification may contribute to left ventricular outflow tract obstruction. Liquefactive degeneration of caseous mitral annular calcification may represent an embolic source. Persistent symptoms or embolic complications require recognition of structural mechanisms.

## Visual Summary

**Figure:**
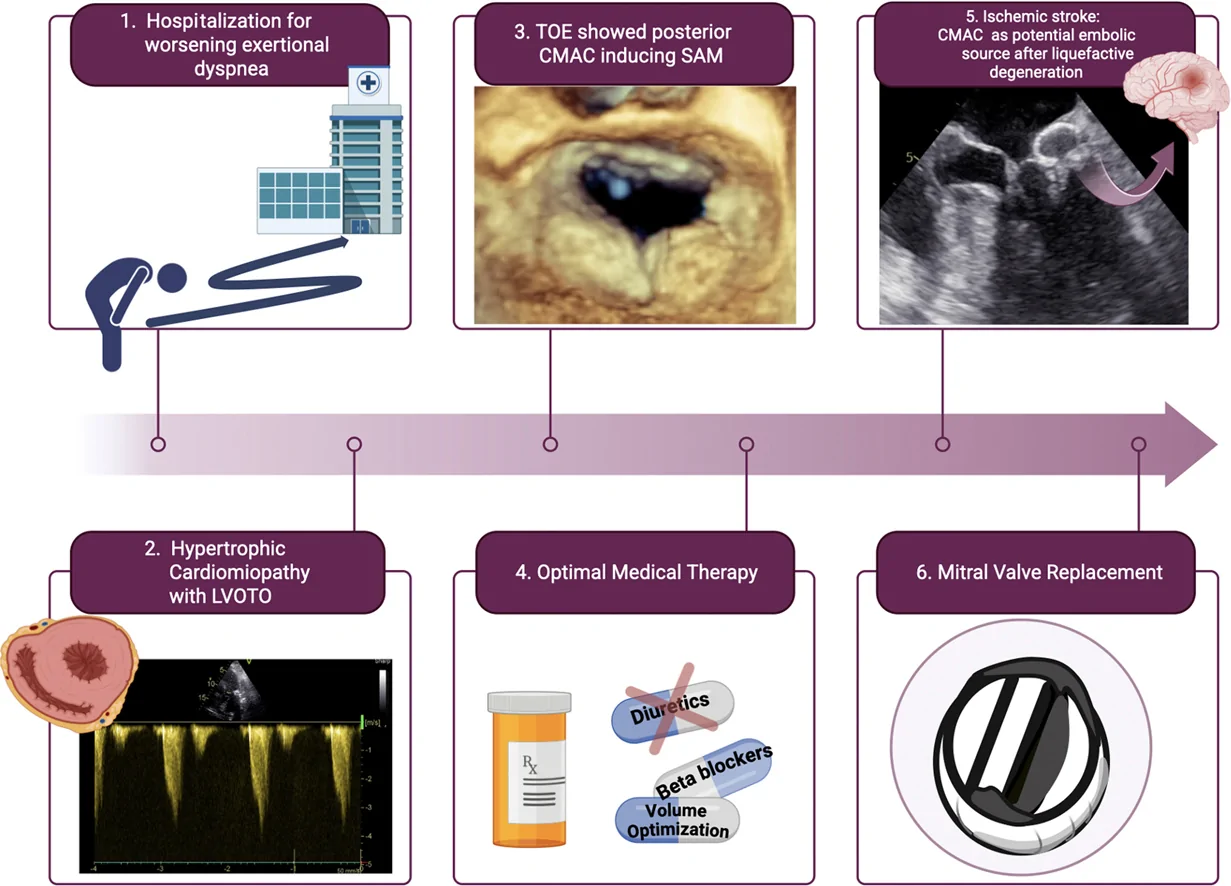

## History of Presentation

A 50-year-old woman presented to the emergency department with a 3-month history of progressively worsening exertional dyspnea (NYHA functional class III). On admission, she was hemodynamically stable, with sinus bradycardia (heart rate, 45 beats/min), normal oxygen saturation, and no clinical signs of overt heart failure. Physical examination was unremarkable.

**Take-Home Messages**

• Caseous mitral annular calcification may contribute to left ventricular outflow tract obstruction by altering mitral valve geometry and promoting systolic anterior motion.
• Liquefactive degeneration of caseous mitral annular calcification may represent a potential embolic source and should be considered in patients presenting with ischemic stroke.
• Persistent symptoms despite optimal medical therapy or embolic complications should prompt early recognition of structural mechanisms and consideration of surgical intervention.

## Past Medical History

Her medical history included arterial hypertension, smoking, overweight, and an anxiety disorder; she had no prior cardiac disease.

**Visual Summary:**
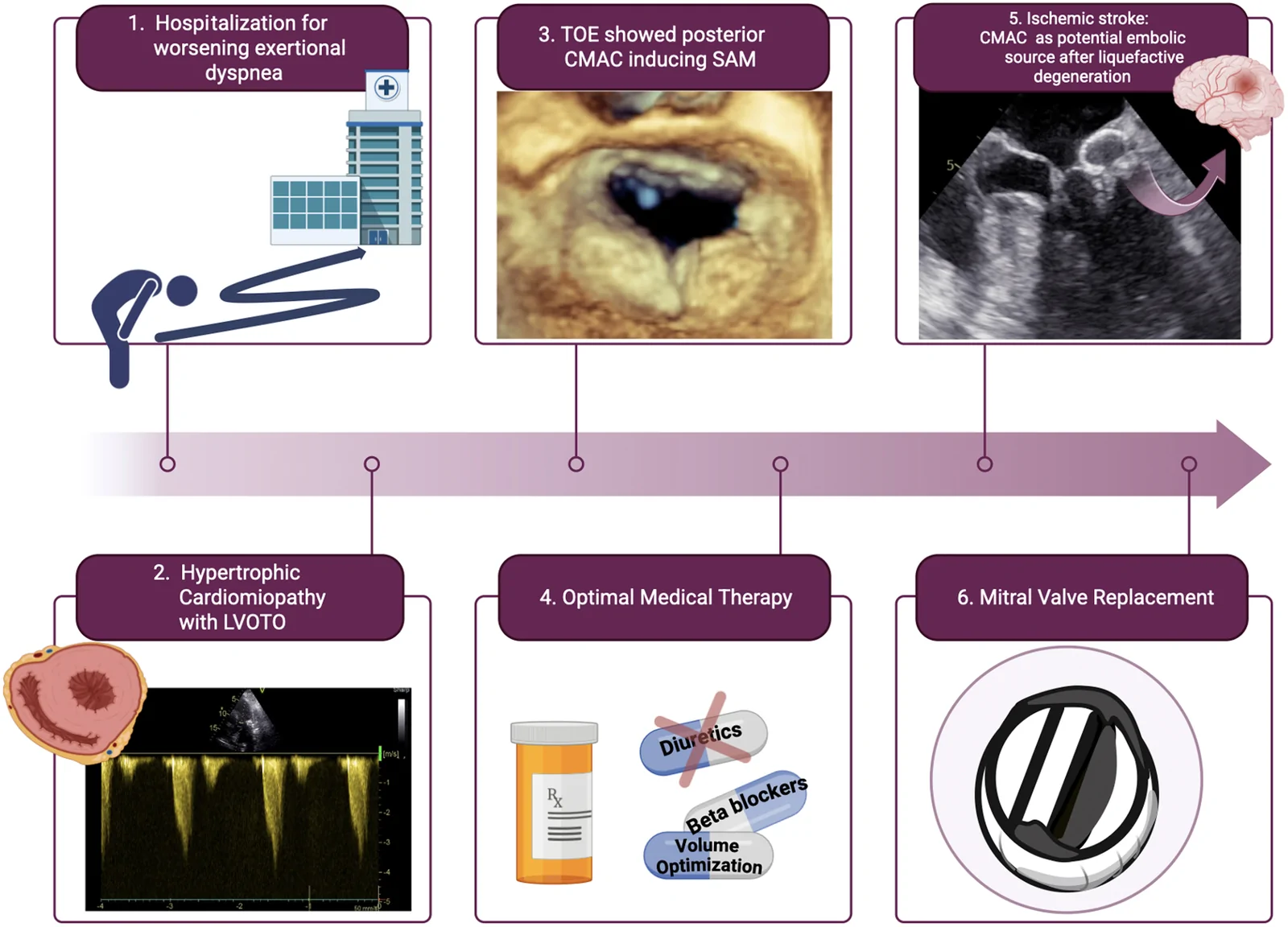
Clinical Course and Illustrating the Progression From Symptomatic Presentation to Multimodality Imaging Findings, Medical Management, Embolic complications, and Definitive Surgical Treatment CMAC = caseous mitral annular calcification; LVOTO = left ventricular outflow tract obstruction; SAM = systolic anterior motion; TOE = transesophageal echocardiography.

## Differential Diagnosis

The differential diagnosis included obstructive hypertrophic cardiomyopathy (oHCM), mitral valve disease, coronary artery disease, and pulmonary embolism.

## Investigations

Computed tomography pulmonary angiography excluded pulmonary embolism. Electrocardiography showed sinus bradycardia with signs of hypertrophy. Chest radiography showed no signs of pulmonary congestion.

Transthoracic echocardiography demonstrated preserved biventricular function with basal septal hypertrophy (15 mm). A dynamic left ventricular outflow tract obstruction (LVOTO) was identified, moderate at rest and reaching a peak gradient of 70 mm Hg after the Valsalva maneuver. The mitral valve showed a huge annular calcification conditioning moderate regurgitation and mild stenosis (mean gradient = 4 mm Hg). Mild aortic regurgitation and mild-to-moderate tricuspid regurgitation with mild pulmonary hypertension (45 mm Hg) were also observed.

**Figure 1:**
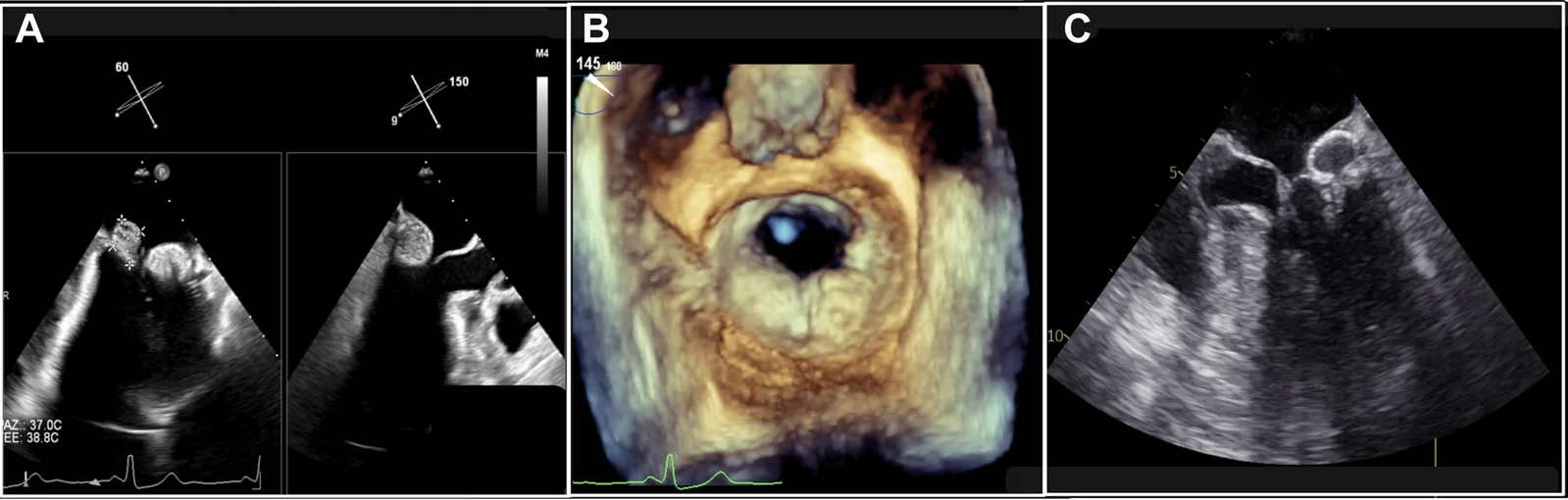
Transesophageal Echocardiography (A) X-plane TEE view of CMAC before vacuolization; (B) 3D TEE view of CMAC; (C) TEE view of CMAC after vacuolization. CMAC = caseous mitral annular calcification; TEE = transesophageal echocardiography.

Transesophageal echocardiography (TOE) provided further anatomical details, revealing 2 large rounded echodense masses located at the mitral annulus (16 × 17 mm and 21 × 19 mm), displaying central echolucency, consistent with caseous necrosis (Video 1). These bulky posterior lesions caused distortion of the mitral annulus and altered leaflet geometry, leading to anterior displacement of the mitral apparatus: this resulted in systolic anterior motion of the anterior leaflet, which was the primary driver of LVOTO, rather than septal hypertrophy alone, and moderate mitral regurgitation, while mitral valve area was only mildly reduced (Figures 1A and 1B, Videos 2 and 3).

**Figure 2:**
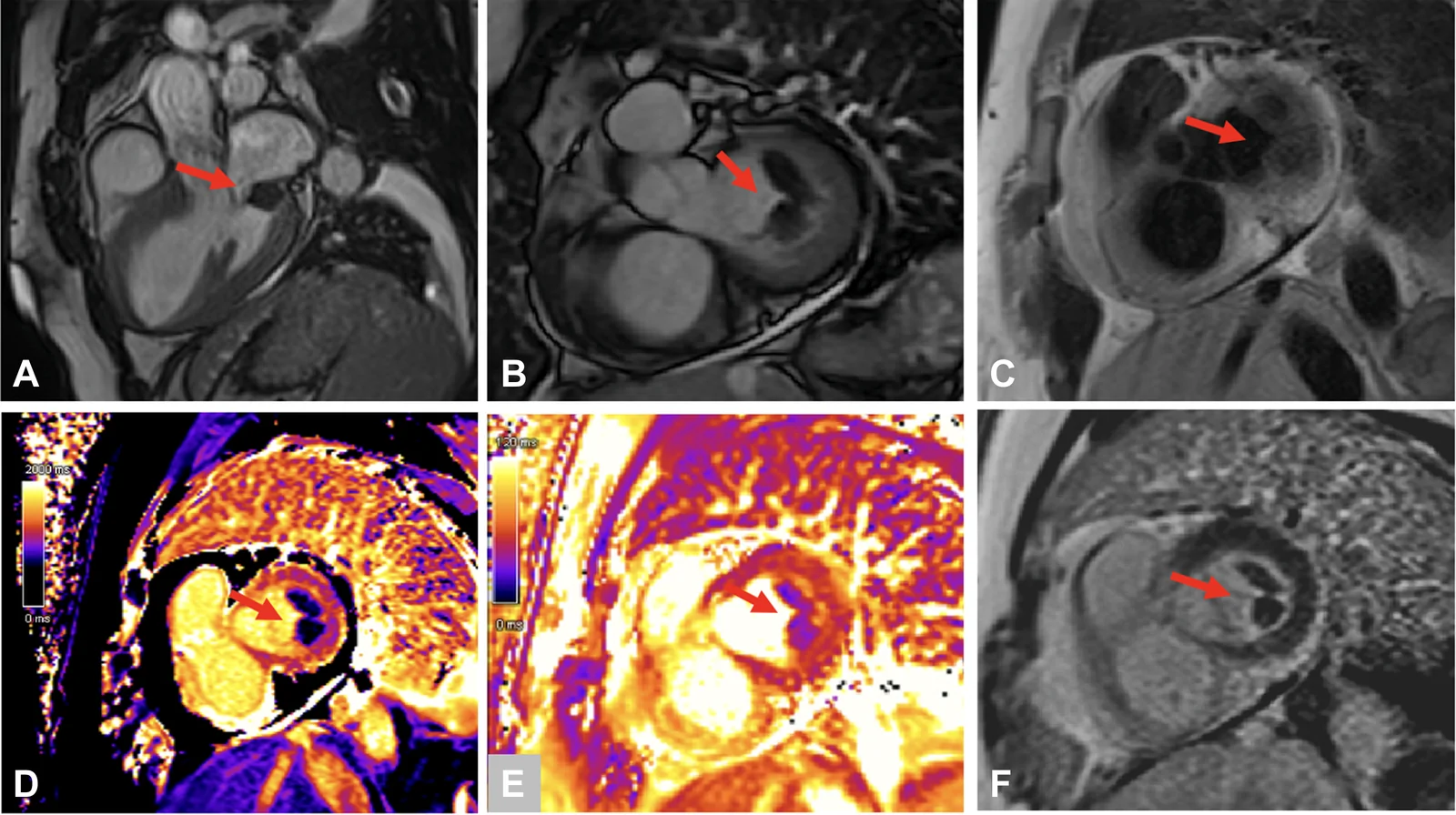
Cardiac Magnetic Resonance Balanced steady-state free precession cine sequences in the 3-chamber view (A) and short-axis view (B) demonstrate a hypointense mass with well-defined borders adjacent to the posterior mitral annulus (red arrow). T1-weighted turbo spin-echo sequence in the short-axis view (C) shows the same hypointense mass (red arrow). On parametric mapping, the lesion exhibits low native T1 and T2 values on T1 mapping (D) and T2 mapping (E), respectively. Phase-sensitive inversion recovery late gadolinium enhancement imaging (F) reveals an enhanced rim surrounding a nonenhanced core.

## Management

After multidisciplinary heart team discussion, the patient was diagnosed with symptomatic oHCM associated with caseous mitral annular calcification (CMAC) and moderate mitral regurgitation. Cardiac magnetic resonance confirmed the septal hypertrophy, with peripheral late gadolinium enhancement of the posterior mitral annulus (Figure 2); storage phenocopies were excluded, including Fabry disease based on laboratory testing. Guideline-directed management was established, with discontinuation of diuretics and introduction of nadolol (80 mg daily). Preexisting lipid-lowering therapy was continued. A stepwise approach was adopted, and priority was given to optimized medical treatment with consideration of mavacamten introduction in case of persistent symptoms, in line with recommendations for oHCM, and reserving surgical intervention for potential treatment failure.

## Outcome and Follow-Up

**Figure 3:**
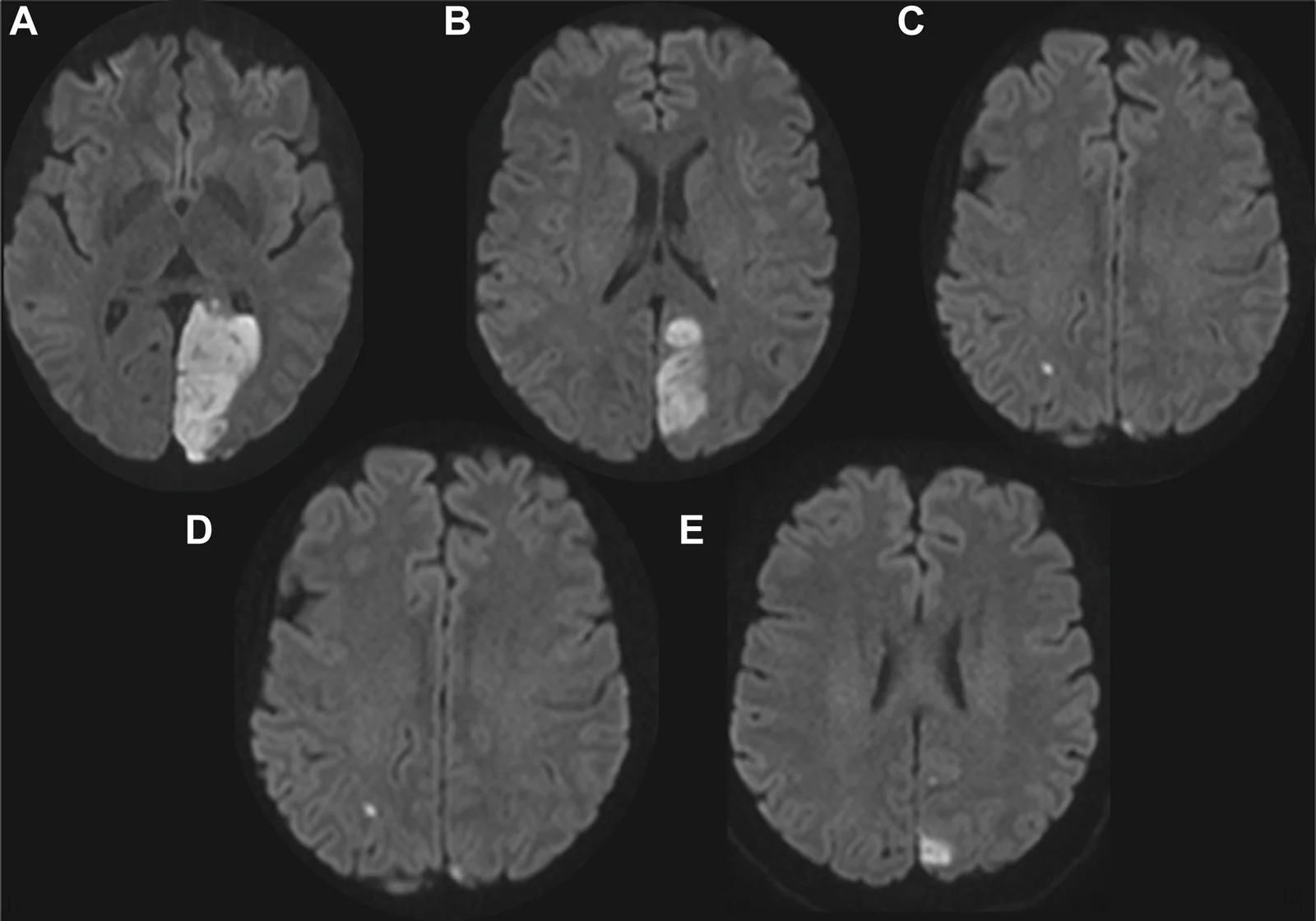
Brain Magnetic Resonance Imaging, Diffusion-Weighted Imaging Sequences (A and B) Large hyperintense area with restricted diffusion consistent with recent ischemic infarction in the left posterior cerebral artery territory, involving the left occipital cortex, subcortical region, and medial parietal hemisphere. (C, D, an E) Additional smaller foci with similar signal characteristics, consistent with recent ischemic lesions, are present in the left hemisphere at the posterior cingulate gyrus and along the lateral wall of the posterior left lateral ventricle. The presence of multiple recent bilateral ischemic lesions involving cortical territories supports an embolic etiology of the stroke.

**Figure 4:**
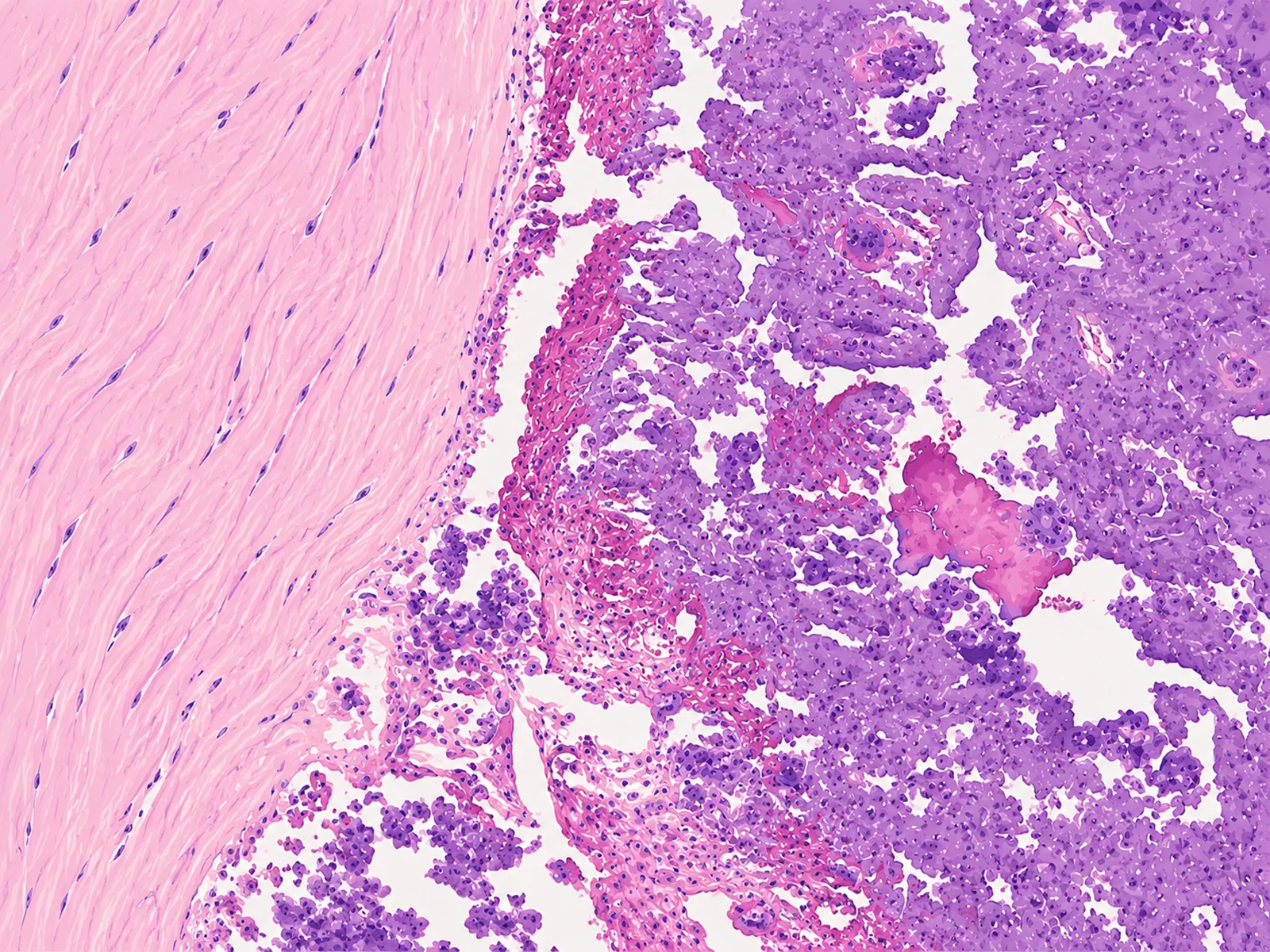
Histologic Findings Demonstrating Hyalinized Tissue With Focal Chronic Inflammation and Focus of Calcification

Two months later, the patient was hospitalized for an ischemic stroke presenting with lateral hemianopsia; brain magnetic resonance imaging revealed recent cerebellar ischemic lesions (Figure 3). Repeat TOE showed no thrombus in the left atrium or in the left atrial appendage, describing features consistent with central liquefactive necrosis involving the known hyperechogenic masses at the posterior mitral annulus (Figure 1C; Video 4). Inflammatory markers were within normal range. Based on these findings, mitral valve replacement was performed to address both mechanical distortion of the valve and resulting LVOTO, as well as to eliminate the suspected embolic source. Intraoperative findings confirmed multiple large vacuolated calcific nodules with caseous content involving the posterior mitral annulus and extending into the adjacent atrioventricular junction myocardium. Histopathologic examination demonstrated mitral valve tissue with fibrosis, focal microcalcifications within the fibrous core, scattered lymphomonocytic infiltrates, and partial fragmentation of the elastic component. At the level of the chordal insertion, a neoformation composed of fibrinoid stratifications with extensive calcifications and adjacent multinucleated giant cells was identified, consistent with advanced degenerative and inflammatory changes of CMAC (Figure 4). A 25-mm mechanical prosthesis was implanted. No postoperative complications occurred, and follow-up echocardiography demonstrated resolution of the dynamic LVOT obstruction. The patient was discharged on nadolol, vitamin K antagonist, and thyroid hormone replacement therapy.

## Discussion

**Figure 5:**
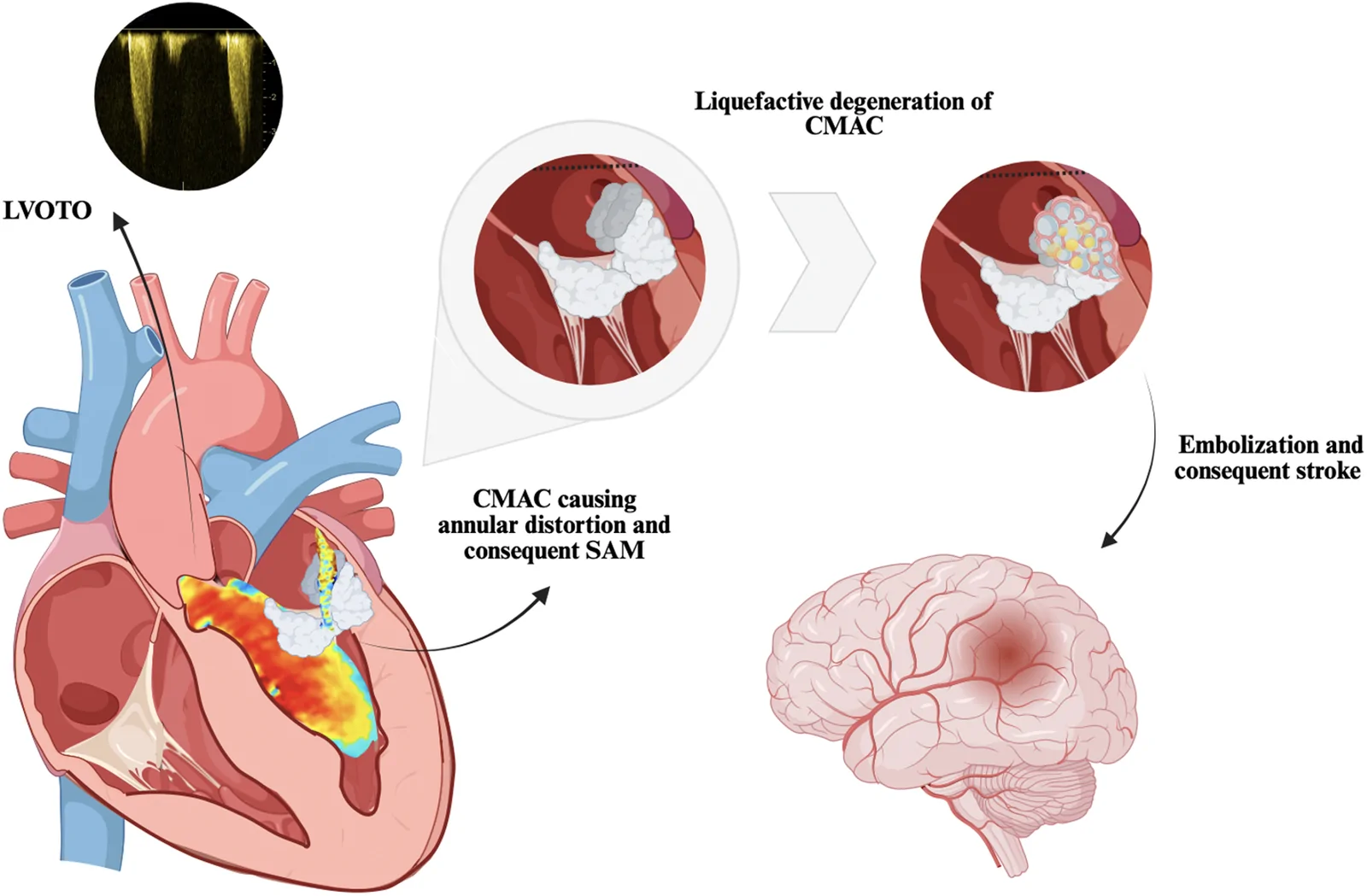
Pathophysiological Relationship Between Mitral Annular Calcification, Hypertrophic Obstructive Cardiomyopathy, and Embolic Events CMAC = caseous mitral annular calcification; LVOTO = left ventricular outflow tract obstruction; SAM = systolic anterior motion.

This case illustrates a rare and clinically relevant association between oHCM and CMAC, with important diagnostic and prognostic implications (Figure 5). CMAC is an uncommon variant of mitral annular calcification characterized by central liquefactive necrosis and may mimic cardiac tumors, abscesses, or thrombi, often leading to diagnostic uncertainty. Beyond its imaging appearance, CMAC may have clinically significant consequences depending on its size, location, and structural evolution.^1^^,^^2^

In this patient, CMAC played a dual pathophysiological role. From a hemodynamic perspective, large posterior annular lesions distorted mitral valve geometry, promoting anterior displacement of the mitral apparatus and facilitating systolic anterior motion, thereby contributing to dynamic LVOT obstruction and mitral regurgitation.^3^ However, a key aspect of this case is the potential embolic risk associated with CMAC. Liquefactive degeneration within the calcified mass may predispose to embolization, and CMAC has been increasingly recognized as a potential source of systemic emboli, including ischemic stroke.^4^ In our patient, the occurrence of stroke raised the suspicion that CMAC may have contributed as an embolic source, further influencing clinical decision-making. Surgical findings further confirmed the presence of extensive caseous degeneration involving the posterior mitral annulus, supporting the hypothesis of a structurally unstable substrate with potential embolic risk.

This case highlights the pivotal role of multimodality imaging not only for diagnostic clarification but also for risk stratification. Although transthoracic echocardiography identified LVOT obstruction and mitral valve dysfunction, TOE was essential to characterize the annular lesions, confirm the diagnosis of CMAC, and suggest its potential instability. Accurate recognition of CMAC is critical to avoid misdiagnosis and to identify patients at increased embolic risk. Management was initially guided by current recommendations for oHCM, including β-blocker therapy and avoidance of preload-reducing agents. Mavacamten represents a novel therapeutic option targeting sarcomeric hypercontractility and may reduce LVOT gradients;^5^ however, in this case, the structural nature of the obstruction and the presence of CMAC-related mitral distortion limited the expected benefit of medical therapy alone. Importantly, the potential embolic risk associated with CMAC, further supported by the occurrence of stroke, represented a key factor prompting consideration of definitive surgical treatment aimed at both relieving LVOT obstruction and eliminating a potential embolic source.

## Conclusions

CMAC can coexist with oHCM and contribute to both dynamic LVOT obstruction and mitral regurgitation through distortion of the mitral valve apparatus. Importantly, CMAC may also represent a potential embolic source, particularly in the setting of liquefactive degeneration. Multimodality imaging is essential for accurate diagnosis and risk assessment, and recognition of both mechanical and embolic implications of CMAC should guide timely multidisciplinary evaluation and consideration of surgical intervention.

## Funding Support and Author Disclosures

Dr Berti is a proctor for Edwards Lifesciences, Abbott, and Boston Scientific. All the other authors have reported that they have no relationships relevant to the contents of this paper to disclose.

## Appendix

Video 1 Three-Dimensional Transesophageal Echocardiographic View of the Mitral Valve Showing Extensive Annular Calcification of the Posterior Annulus

Video 2 Transesophageal X-Plane View of the Mitral Valve Color Doppler shows acceleration in the left ventricular outflow tract caused by anterior displacement of the mitral leaflets and consequent systolic anterior motion.

Video 3 Transesophageal 4-Chamber View Demonstrating Systolic Anterior Motion of the Mitral Valve

Video 4 Poststroke Transesophageal X-Plane View of the Mitral Valve A vacuolated appearance of mitral annular calcification is noted.
